# Cultivating Health: The Role of Urban Greening in Supporting Baltimore’s Youth

**DOI:** 10.1007/s11524-025-01022-6

**Published:** 2025-11-03

**Authors:** Kristin Mmari, Marina C. Jenkins, Rebecca Skinner, Beth Marshall, Cara Wychgram, Dustin Fry, Dexter H. Locke, Amanda Phillips-De Lucas, Michelle C. Kondo

**Affiliations:** 1 Department of Population, Family, and Reproductive Health, Johns Hopkins Bloomberg School of Public Health, 615 North Wolfe Street, Baltimore, MD 21205 USA; 2 Spatial Science for Public Health Center, Johns Hopkins Bloomberg School of Public Health, Baltimore, MD USA; 3 USDA Forest Service Northern Research Station, Philadelphia Field Station, Philadelphia, PA USA; 4 USDA Forest Service Northern Research Station, Baltimore Field Station, Baltimore, MD USA; 5 University of Baltimore, Baltimore Neighborhood Indicators Alliance—Jacob France Institute (BNIA-JFI),, MD USA

**Keywords:** Adolescents, Urban health, Green space, Mixed methods

## Abstract

Project VITAL (Vacant Lot Improvement to Transform Adolescent Lives) is a study designed to evaluate the impact of revitalized vacant lots on adolescent health in Baltimore, Maryland. We implemented a mixed-methods research approach, which included surveys of 14- to 19-year-olds, observations of nearby vacant lots, and street block assessments during 2023–2024. The aim was to understand how greening vacant lots affected adolescent mental health, experiences of violence, and food insecurity. Linear regression models were used to examine the association between living near a maintained vacant lot and various outcomes. Effect size was measured for each significant association using Cohen’s* f*^2^*.* Out of the 313 survey participants with geo-coded addresses, 50.2% resided within 0.20 mi of a maintained lot. Proximity to these maintained green spaces was associated with greater happiness (*p* = 0.01, *f*^2^ = 0.04) and reduced food insecurity among adolescents, although the latter did not achieve statistical significance (*p* = 0.08, *f*^2^ = 0.06). No significant link was found between the proximity to green spaces and either depressive symptoms or experiences of weapon-related violence. These findings highlight the complexity of the effects of urban greening, indicating that while it may not address all negative outcomes, it can modestly improve certain positive aspects of adolescent well-being. Public health initiatives focusing on these efforts could help address urban decay and promote long-term health equity.

## Introduction

Vacant lots are a common feature in urban environments, particularly in post-industrial regions of the USA, where a decline in population over the past 50 years has resulted in housing vacancies and declining infrastructure [1–3]. Adolescents growing up in such conditions face heightened risks of poor mental and physical health outcomes [4, 5]. However, transforming vacant lots into maintained green spaces, including community gardens and spaces for recreation, offers a promising solution for enhancing community well-being and is increasingly being pursued as an urban planning strategy to advance public health, particularly in underserved communities [6, 7]. This approach is predicated on multiple benefits that green spaces can offer, such as increased physical activity opportunities [8], improved air quality [9], mental health enhancements [10], reduced experiences of violence [11], and food insecurity [12]. A growing body of research has already established the crucial impact of neighborhoods on adolescent health, illustrating how the social and physical environments young people inhabit influence their access to resources, social support, and opportunities for healthy living [13, 14]. Safe and cohesive neighborhoods can contribute to better health among adolescents by fostering a sense of belonging and community, which can alleviate the stressors linked to socioeconomic disadvantage. For adolescents, who undergo significant physical, social, and emotional development, the health benefits of urban greening projects in their neighborhoods can, therefore, be especially profound.

Assessing the exact impact of greening vacant lots on adolescent health, however, involves numerous methodological challenges. Implementing experimental and quasi-experimental study designs is often impractical, particularly in urban areas where simultaneous exposure to both maintained and unmaintained vacant lots complicates the creation of a control group and when no reliable inventory of vacant lots or records of their maintenance exists. Since greening efforts can encompass a variety of activities—from mowing grass and removing trash to establishing community gardens and creating art murals—it is essential for any study design to account for these differences to accurately determine the health impacts [15].

Baltimore City is currently grappling with over 20,000 vacant lots and an additional 17,000 abandoned buildings [16]. In response, the Baltimore Office of Sustainability developed the Green Network Plan, aiming to “clean and green” vacant lots in neighborhoods with high vacancy rates [17]. While more than 900 lots have been greened so far, the methods and maintenance of these spaces vary significantly, and comprehensive records of these actions did not exist before our study. Some greening efforts focus on mowing and trash pickup, while others incorporate art murals, community gardens, and host community programs and events. We launched Project VITAL (Vacant Lot Improvement to Transform Adolescent Lives) [7, 18] in 2020 to inventory and characterize these diverse greening efforts and assess their impacts on adolescent health in Baltimore. This paper presents the initial study findings, emphasizing the relationship between living near a maintained greened vacant lot and adolescent mental health, experiences of violence, and food insecurity.

## Methods

### Overview

**Fig. 1:**
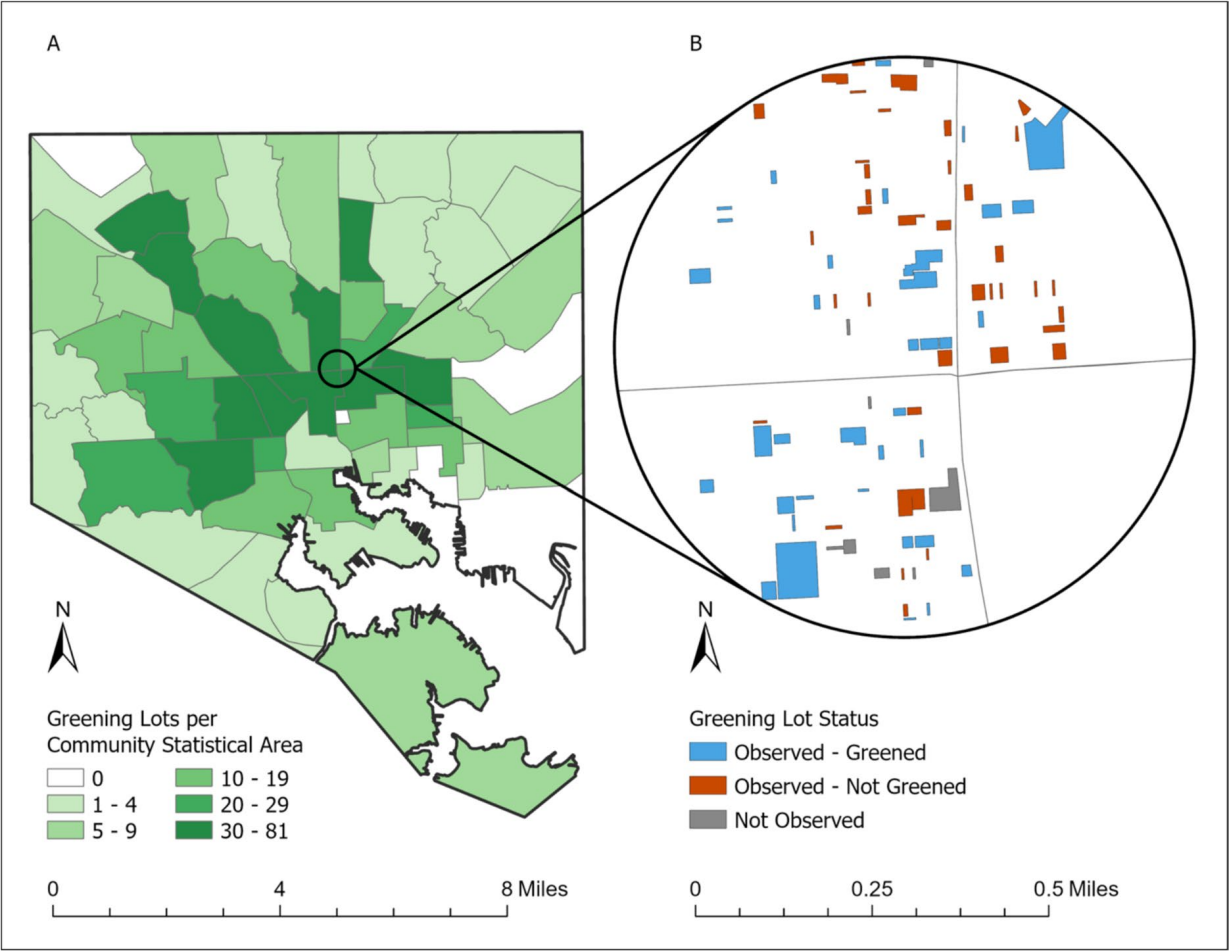
Distribution of greened vacant lots in Baltimore City

This mixed-methods study included (1) surveys with 14–19-year-olds in Baltimore, (2) observations of vacant lots within 0.20 mi of survey participants’ homes, and (3) a street block assessment to evaluate the built environment around the lots (Fig. 1). In addition, we calculated potential area-level confounders of the relationship between the presence of greened vacant lots and adolescent health, including neighborhood-level poverty rates and block-level physical disorder scales. Neighborhood-level poverty was calculated by residential addresses using publicly available data on the percent of residents below the poverty line by Community Statistical Area, obtained by the Baltimore Neighborhood Indicator Alliance. Block-level physical disorder was measured by a score based on five binary items from the street-block assessment tool to allow comparison between blocks of various lengths. Items include the presence of broken windows, abandoned buildings, trash, graffiti, and landscaping (reverse-coded). A confirmatory factor analysis (CFA) estimated a perfect model fit (root-mean-square error of approximation [RMSEA] of 0 and comparative fit index [CFI] of 1).

Ethical approval was obtained from the Johns Hopkins Bloomberg School of Public Health Institutional Review Board (#21,175).

### Adolescent Surveys

Surveys were conducted among adolescents at in-person events in collaboration with community partners from October 2022 until September 2023. Adolescents were eligible for the survey if they lived in a Baltimore City zip code and were aged between 14 and 19 years, which was obtained during the consent and assent procedures. We received adolescents’ signed consent if they were 18 years and above; for adolescents younger than 18, we obtained their written assent and parent/guardian permission orally over the phone. The survey was collaboratively designed by the Project VITAL team and a youth advisory board consisting of five adolescents aged 14–19 years. Adolescents completed the self-administered surveys using tablets in private outdoor locations and were asked questions about sociodemographic characteristics, perceptions of their neighborhood and accessibility to green spaces, and health outcomes such as symptoms of depression, happiness, experiences with weapon violence, and food insecurity. These constructs were selected based on previous research on greening vacant lots among adults [10], as well as our research that has examined the associations between adolescent food insecurity and the neighborhood [19].

### Survey Measures

#### Outcomes

We collected two different indicators of mental health status that the characteristics of nearby vacant spaces could influence. We assessed symptoms of depression among participants, as vacant lot greening has been shown to reduce symptoms of depression among adults [10]. *Depression* was measured using the 10-item Center for Epidemiologic Studies Depression Scale (CES-D-10) [20]. Participants were asked to indicate how they felt or behaved in the past week on a scale from 0 (< 1 day) to 3 (5–7 days), including *I felt lonely*. The depression score is a sum of all items, ranging from 0 to 30, with higher scores indicating greater depressive symptomology. We also measured *happiness* using the EPOCH Measure of Adolescent Well-Being Happiness subscale, as a positive measure of adolescent mental health that may be influenced by access to neighborhood green space [21]. Respondents indicated agreement with statements on a scale of 1 (not at all like me) to 5 (exactly like me), including *I am a cheerful person*. The happiness score was calculated as an average rating across four items, with a possible range of 1–5. Validation in our sample estimated a perfect model fit (RMSEA of 0 and CFI of 1).

*Experience of weapon violence* was measured as a sum of affirmative responses to seven questions regarding lifetime experiences of violence with a weapon, including witnessing and victimization, ranging from 0 to 7. Items were adapted from the Screen for Adolescent Violence Exposure (SAVE) [22] and include *I have been shot* and *I have seen someone attacked with a knife*.

*Food insecurity* was measured using a validated nine-item tool adapted from the US Food Security Survey Module [23]. Items include, for example, “In the past 30 days, how often were you hungry but didn’t eat because your family didn’t have enough food?” Food insecurity status is intended for dichotomous grouping based on the number of affirmative responses: food secure (0–1) and food insecure (2–9).

*Sociodemographic characteristics* were obtained from survey responses*:* age, race/ethnicity, gender, household size, and housing instability. Age was categorized into three groups (14, 15–16, 17–19) to reflect the differences in food insecurity that are often reported between younger and older children [24]. Household size was characterized as < 4 members, 4 members, or > 4 members to align with the USDA’s Thrifty Food Plan [25]. Housing instability was a binary measure based on affirmative responses to frequent changes in or concerns about losing stable sleeping arrangements.

#### Individual-Level Covariates

*Neighborhood social cohesion* was measured by ten items drawn from previous studies [26]. Following confirmatory factor analysis in our sample, we identified a three-factor model for neighborhood social cohesion with a good model fit with a CFI of 0.99 and an RMSEA of 0.06. Three variables were derived from the three factors: familiarity (three items), including “In your neighborhood, how many adults would you say you recognize?”; informal policing (four items), including “How likely is it that your neighbors would report children showing disrespect to an adult to their parents?”; and relationships between adults and children (three items), including “How much do you agree or disagree with this statement: In your neighborhood, adults take the ideas of youth seriously?” Respondents indicated agreement with each item on a 5-point Likert scale from 0 to 4, and items were summed for each factor, with higher scores indicating greater social cohesion.

*Perceived neighborhood safety* was measured using six questions adapted from a previous study, including “How fearful of crime are you in your neighborhood?” [27]. Responses were summed across all questions to create an overall safety score, ranging from 0 to 20, with higher values indicating lower perceived levels of neighborhood safety. CFA in our sample showed an acceptable fit for a single-factor scale, including all items (CFI = 0.98, RMSEA = 0.09) and internal consistency (0.78).

*Perceived neighborhood disorder* was measured using an eight-item scale to capture adolescent perspectives on neighborhood conditions [26]. Participants indicated agreement with statements using a 5-point Likert scale, with higher values indicating greater disorder, including “There is a lot of graffiti.” Responses were summed across all questions to create a perceived neighborhood disorder score, ranging from 0 to 32. CFA in our sample showed an acceptable fit for a single-factor scale, including all items (CFI = 0.98, RMSEA = 0.09) and internal consistency (omega value = 0.82).

*Post-traumatic stress disorder (PTSD)* was measured using the four-item, short-form PCL-5 [28]. We selected this as a covariate as previous research has shown associations with weapon violence [29]. Participants were asked to identify how bothered they were by the item on a scale from 0 (not at all) to 4 (extremely), including *feeling jumpy or easily startled*. The PTSD score is the sum of all items, ranging from 0 to 16.

*Hope* was measured using three items derived from the Urban Adolescent Hope Scale [30]. Respondents were asked to rate their agreement that the statement described themselves on a scale from 1 (not at all like me) to 5 (exactly like me), including “I am excited about my future.” Items were summed, with a score ranging from 3 to 15. This was selected as a covariate as previous research has shown the relationship between hope and violence [31].

### Observations

*Greened vacant lot observations:* Greened vacant lots were identified as sites consisting of one or more parcels that have a history of stewardship and greening activities. A comprehensive database of greened vacant lots in Baltimore City (*n* = 871) was developed in collaboration with the Baltimore Neighborhood Indicator Alliance, which gathered greening data from city- and community-based organizations involved in the stewardship of vacant lots from 1911 to 2022.

For this study, 456 greened vacant lots were selected for in-person observations based on being within a 0.20-mi radius of a participant’s home, consistent with previous studies on green spaces [32]. Survey data and lot observation data were linked at the participant level, allowing each lot to be associated with multiple participants. The observation tool was adapted from existing tools measuring lot features, quality, and type of greening activities implemented (e.g., community garden, flower beds) using both closed-ended and open-ended questions^33^. Observations were done in pairs, including one young person from a local community-based organization and one university graduate student. Each observer completed the tool independently on a tablet. We also used observations to confirm whether greened vacant lots were located at the correct addresses.

Greened vacant lots were classified as maintained or not maintained based on ratings of observed greening and overall quality averaged between observers. The greening scale was composed of the quantities of grass, trees, and garden plots, plus the presence of murals. The quality scale included the quantity of trash, abandoned buildings, perceived maintenance, and condition of structures. We performed CFA, finding an excellent model fit for both scales (greening: CFI = 0.99, RMSEA = 0.01; quality: CFI = 0.99, RMSEA = 0.06). Lots that met a threshold score of three or higher for greening, indicating complete coverage by vegetation, and 7.9 for quality, reflecting the mean quality of all observed lots, were classified as maintained. Figure 1 depicts the distribution of all greened vacant lots across the city. The zoomed-in area on the right depicts an example of how lots were characterized as observed and maintained, observed and not maintained, or not observed.

*Street-block observations:* Observations were conducted for the street blocks that survey participants lived on in November 2023 to capture objective conditions of survey respondents’ immediate environment. The unit of observation was determined by using the participant’s home address and rounding down to the nearest 100th level of the block for standardization. For example, if a participant lived at 2160, the block that was observed was from 2100 to 2199. The observation tool was adapted from an existing tool [33] on neighborhood conditions, and observations were conducted using the same methods as lot observations.

**Fig. 2:**
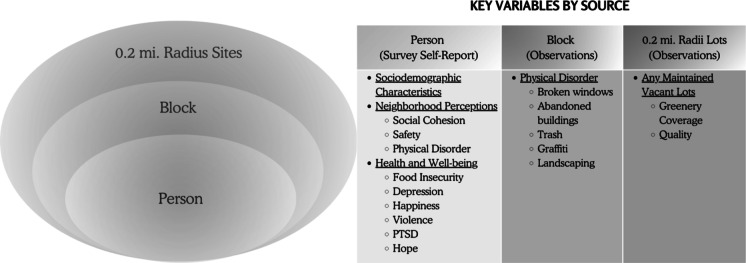
Person-centered data configuration by levels of spatially-nested data collection

Figure 2 illustrates the type of data collected by surveys, greened lot observations, and street-block observations.

### Statistical Analysis

The unit of analysis for this study was survey participants. Observation data for 247 residential city blocks and 456 greened vacant lots were linked to survey data. We first ran descriptive statistics of our independent and dependent variables overall and by exposure status. We then ran bivariate linear regression models between the dependent variable and each of the outcomes of interest and determined relevant covariates.

Finally, we ran multi-level generalized linear regression models with robust standard errors to test associations between exposure to at least one maintained greened vacant lot within 0.20 mi of residence and adolescent outcomes. Regression models were clustered by residential city blocks to account for block-level spatial dependence and non-independence of block and vacant lot observation data. Additionally, residuals from each model were tested for spatial dependence using semivariogram plots and for multicollinearity by calculating variance inflation factors (VIF), and no evidence of spatial dependence or multicollinearity was identified for any outcome. Moreover, we did not find evidence of collinearity between any considered covariates, based on |*r*|< 0.75. Models were also run with a continuous independent variable for a count of maintained lots within 0.20 mi of a respondent’s residence. Age, gender, and CSA-level poverty rates were considered as potential covariates for all regression models; additional covariates varied by outcome and were selected based on theoretical relevance. Since there is no existing evidence on which of these variables were likely to be confounders, those that were not significantly associated with the outcome in bivariate models at the *p* < 0.10 level were excluded [34]. We retained household size in the food insecurity model based on its relevance for food assistance program qualification. Interaction terms between the primary exposure and each covariate were also used to test effect modification. Effect size was measured for each significant association using Cohen’s* f*[235].

Associations with *p* < 0.05 were considered statistically significant. Analyses were conducted in Stata 17.0 [36].

## Results

Our study included 313 adolescent survey respondents with an average age of 15.6 (SD = 1.7), of whom 50.2% identified as female and 74.8% identified as African American (Table 1). Participants resided in 25 unique zip codes within Baltimore City and 247 unique street blocks. Of the 456 observed lots, 48.9% (*n* = 223) were classified as maintained. Approximately half of the participants (50.2%) lived within 0.20 mi of at least one maintained greened vacant lot, and 43.1% of participants lived within 0.20 mi of at least one unmaintained greened vacant lot.

**Table 1:** Participant characteristics by maintained greened vacant lot exposure (*N* = 313)

Variable	Full sample		Any maintained lot exposure			
	(***N*** = 313)		No (***n*** = 156)		Yes (***n*** = 157)	
	***n***	%	***n***	%	***n***	%
Gender						
Male	146	47	76	49	70	45
Female	157	50	76	49	81	52
Other	10	3	4	3	6	4
Age group						
14	106	34	56	36	50	32
15–16	117	37	54	35	63	40
17–19	85	27	44	28	41	26
Race/ethnicity						
African American (NH)	234	75	113	72	121	77
Multiple/other (NH)	24	11	15	10	19	12
Hispanic	27	9	17	11	10	6
White (NH)	18	6	11	7	7	4
Highest grade						
8th grade	82	26	43	28	39	25
9th grade	84	27	39	25	45	29
10th grade	49	16	17	11	32	20
11th grade	35	11	20	13	15	10
12th grade or equivalent	51	16	26	17	25	16
Some college or technical	11	4	10	6	1	1
Household size						
< 4	123	39	65	42	58	37
4	85	27	41	26	44	28
> 4	105	34	50	32	55	35
Housing instability	38	12	23	15	15	10
Food insecurity	138	44	78	57	60	44
**Variable**	**Mean**	**SD**	**Mean**	**SD**	**Mean**	**SD**
Neighborhood-level poverty percent	20	10.85	18	11.20	22	10.05
PTSD score	5	4.02	5	3.94	5	4.12
Perceived neighborhood disorder	15	5.22	14	5.16	16	5.08
Perceived neighborhood safety	9	4.38	9	4.25	10	4.43
Neighborhood cohesion—familiarity	5	2.30	6	2.20	5	2.38
Neighborhood cohesion—informal policing	8	3.72	8	3.62	8	3.81
Neighborhood cohesion—relationships	6	2.47	6	2.37	6	2.57
Neighborhood physical disorder	2	1.59	2	1.34	3	1.59
Hope score	11	3.24	11	3.11	11	3.36
Depression score	10	5.65	10	5.50	10	5.80
Happiness score	12	2.88	12	2.89	13	2.86
Weapon violence experiences	2	2.22	2	2.27	2	2.18

*SD* standard deviation, *NH* non-Hispanic

### Depression

The average depression symptom score was 9.9 (SD = 5.7)*.* The final linear regression model for the relationship between depression score and living within 0.20 mi of at least one maintained lot was adjusted for gender, neighborhood cohesion, food insecurity, and neighborhood-level poverty, with no significant interactions. Adolescents with maintained greened vacant lot exposure did not have a statistically significant difference in depression score compared to those without maintained lot exposure (β = 0.11. *p* = 0.85; Table 2)*.*

**Table 2:** Generalized linear regressionmodels between exposure to maintain greened vacant lot and adolescent health outcomes

Outcome	Unadjusted models			*p*-value	Adjusted models			
	Coefficient	95% confidence interval			Coefficient	95% confidence interval		***p***-value
Depression^a^ (*n* = 282)	0.00	− 1.34	1.35	1.00	0.11	− 1.02	1.24	0.85
Happiness^b^ (*n* = 282)	0.44	− 0.26	1.13	0.22	2.25	0.53	3.96	0.01
Weapon violence^c^ (*n* = 285)	− 0.01	− 0.55	0.53	0.97	− 0.17	− 0.66	0.33	0.51
Food insecurity^d^ (*n* = 294)	− 0.56	− 1.35	0.24	0.17	− 0.10	− 0.22	0.01	0.08

^a^Adjusted for age, safety score, and household size

^b^Adjusted for gender, social cohesion - relationships, food insecurity, poverty

^c^Adjusted for gender, social cohesion - familiarity and relationships, food insecurity, physical disorder

^d^Adjusted for PTSD score, food insecurity, hope score, neighborhood physical disorder, and perceived neighborhood disorder

### Happiness

The average happiness score was 12.2 out of 16 (SD = 2.9). The final linear regression model for the relationship between the happiness score and living within 0.20 mi of at least one maintained lot was adjusted for gender, neighborhood cohesion, food insecurity, and neighborhood physical disorder, with a significant interaction between neighborhood cohesion (familiarity). Adolescents with maintained greened vacant lot exposure had a significantly higher happiness score of 2.25 (95% CI: 0.53, 3.96) compared to those without maintained lot exposure (*p* = 0.01; Table 2)*.* Average marginal effects suggest that familiarity was significantly and positively associated with happiness among adolescents without maintained greened vacant lot exposure (β = 0.24, *p* = 0.01) but not the exposed group (β =  − 0.05, *p* = 0.65). The effect size for this relationship was a Cohen’s *f*^2^ value of 0.04, which is considered a small effect based on standard recommendations [35].

### Weapon Violence

Participants reported an average of 1.86 (SD = 2.2) types of experiences with weapon violence out of seven*.* The final linear regression model for the relationship between total weapon violence experiences and living within 0.20 mi of at least one maintained lot was adjusted for PTSD, food insecurity, hope, neighborhood physical disorder, and perceived neighborhood disorder, with no significant interactions. Adolescents with maintained greened vacant lot exposure did not have a statistically significant difference in reported weapon violence experiences compared to those without maintained lot exposure (β =  − 0.66, *p* = 0.51; Table 2)*.*

### Food Insecurity

The prevalence of food insecurity was 44.1%. The final linear regression model for the relationship between food insecurity and living within 0.20 mi of at least one maintained lot was adjusted for age, perceived safety, and household size, with no significant interactions. Adolescents with maintained greened vacant lot exposure had a 10.0% (95% CI: − 22.0%, 0.10%) lower level of food insecurity compared to those without maintained lot exposure, which neared statistical significance (β =  − 0.10, *p* = 0.08; Table 2)*.* The effect size for this relationship was a Cohen’s *f*^2^ value of 0.06, which is considered a small effect based on standard recommendations [35].

## Discussion

The initial findings from Project VITAL offer valuable insights into the potential health and well-being benefits of living near maintained greened vacant lots for adolescents, who are at a critical stage of physical, social, and emotional development. Overall, the study underscores that the complex relationships between transforming vacant lots into green spaces in disadvantaged neighborhoods can influence adolescent health. Unlike previous research on the associations between green space and adolescent mental health, our study did not find any association between depressive symptoms and living next to a maintained greened vacant lot. However, we did find a small but significant positive association with adolescent happiness. This may suggest that while greened vacant lots might not directly mitigate symptoms of clinical mental health problems, they could enhance overall adolescent well-being [37].

The link between maintained vacant lots and happiness is particularly intriguing. Recently, it has been recognized that mental health encompasses not just the absence of psychological symptoms but also the presence of positive psychological states [38, 39]. This recognition underscores the importance of exploring positive mental health outcomes, such as happiness, to better understand how to improve the quality of life for adolescents, especially those living in disadvantaged neighborhoods. This growing awareness may also be driven by urgent calls from the World Health Organization (WHO) and the United Nations to incorporate positive well-being indicators into policymaking. However, it is only recently that happiness, as a positive aspect of mental health, has been studied in relation to adolescent health. For example, a study using data from Add Health, a nationally representative cohort study of US adolescents, revealed that a higher positive affect during adolescence was linked to better physical and mental health in adulthood [40]. A longitudinal study found that psychological assets in adolescence, including happiness, had the strongest associations with positive cardiovascular health among Black participants [41]. Thus, greening vacant lots could be considered a viable health-promoting strategy to enhance adolescent well-being and health equity. While we did not measure or test mechanisms of association, it is possible that by reducing exposure to urban decay and providing spaces for physical activity and social interaction, greened lots may help protect adolescents from the negative impacts of urban stressors typically found in areas with high vacancy and abandonment rates [42].

Our study identified a potentially significant link between proximity to well-maintained greened vacant lots and reduced food insecurity among adolescents. This near-significant relationship suggests that access to green spaces may enhance food security within local communities. The benefits of these spaces likely arise from higher levels of community cohesion and the promotion of urban agriculture initiatives, such as community gardens, which can serve as a buffer against food scarcity in resource-limited neighborhoods. Notably, vacant lots are often situated in areas with limited access to high-quality green spaces, particularly in socioeconomically disadvantaged communities. Research by Daundasekara et al. [43] demonstrated that higher perceived collective efficacy within neighborhoods can facilitate informal support mechanisms that address the nutritional needs of at-risk families. Their findings indicate that collective efficacy can alleviate food insecurity by fostering informal, instrumental support within the community, effectively creating a “reciprocal economy” that helps meet these needs. Additionally, Denney et al.[44] underscore the protective effect of perceived social cohesion against food insecurity, particularly for racial and ethnic minority households that are disproportionately affected by food scarcity. Their conclusions further suggest that higher levels of neighborhood social cohesion can mitigate the risk of food insecurity, highlighting the critical role of community connections in addressing this issue and advocating for community-focused interventions that strengthen social networks [44].

Interestingly, our study did not find an association between adolescent experiences of weapon violence and greened vacant lot exposure, which contrasts with other research that has shown a significant relationship [45, 46]. One explanation is that while green spaces can enhance perceived neighborhood safety, they alone might not suffice to decrease actual violence experiences among adolescents. Comprehensive community strategies that incorporate greening with additional safety and social interventions could be more effective in reducing violence exposure.

Our study has several limitations. First, the cross-sectional nature of our study precludes causal inference, and the reliance on self-reported measures introduces potential biases. In addition, of our original survey sample of 364, 51 (14.0%) were excluded from this analysis due to missing or unreliable residential addresses. Those who were excluded, however, did not vary significantly by any key demographic variables, including housing instability. Individuals who did not live by any greened vacant lots or who lived only next to unmaintained lots were grouped in our unexposed group; however, we did not find significant differences between these groups for our outcomes. This suggests that maintenance may be a key mechanism for the impact of greening vacant lots on adolescent well-being. Additionally, the variability in urban greening interventions and their maintenance across different sites highlights the necessity for standardized definitions and practices within urban greening initiatives. This standardization is essential for accurately isolating their effects on health outcomes. Future research should address these variations to deepen our understanding of effective greening strategies. Finally, this study assumes that adolescents primarily access maintained greened vacant lots within their residential neighborhoods. However, this assumption may not hold true for Baltimore, where many adolescents attend high schools outside their local areas. Thus, further research is needed to assess whether the exposure to greened vacant lots is greater in residential neighborhoods compared to those associated with schools or peers.

## Conclusions

Greening vacant lots holds significant promise for enhancing adolescent and community health and well-being. As urban planners consider approaches to address vacant buildings and lots, integrating green space development is a strategy that can not only help stabilize neighborhoods but may also improve health for young people. Continued investment in and evaluation of these initiatives can aid in maximizing their impact and sustainability.
